# Review of consensus test methods in medical imaging and current practices in photoacoustic image quality assessment

**DOI:** 10.1117/1.JBO.26.9.090901

**Published:** 2021-09-11

**Authors:** Jorge Palma-Chavez, T. Joshua Pfefer, Anant Agrawal, Jesse V. Jokerst, William C. Vogt

**Affiliations:** a University of California San Diego, Department of NanoEngineering, La Jolla, California, United States; b Center for Devices and Radiological Health, U.S. Food and Drug Administration, Silver Spring, Maryland, United States; c University of California San Diego, Department of Radiology, La Jolla, California, United States; d University of California San Diego, Materials Science and Engineering Program, La Jolla, California, United States

**Keywords:** phantoms, standardization, performance testing, photoacoustics, ultrasound, magnetic resonance imaging, x-ray computed tomography, quality assurance

## Abstract

**Significance**: Photoacoustic imaging (PAI) is a powerful emerging technology with broad clinical applications, but consensus test methods are needed to standardize performance evaluation and accelerate translation.

**Aim**: To review consensus image quality test methods for mature imaging modalities [ultrasound, magnetic resonance imaging (MRI), x-ray CT, and x-ray mammography], identify best practices in phantom design and testing procedures, and compare against current practices in PAI phantom testing.

**Approach**: We reviewed scientific papers, international standards, clinical accreditation guidelines, and professional society recommendations describing medical image quality test methods. Observations are organized by image quality characteristics (IQCs), including spatial resolution, geometric accuracy, imaging depth, uniformity, sensitivity, low-contrast detectability, and artifacts.

**Results**: Consensus documents typically prescribed phantom geometry and material property requirements, as well as specific data acquisition and analysis protocols to optimize test consistency and reproducibility. While these documents considered a wide array of IQCs, reported PAI phantom testing focused heavily on in-plane resolution, depth of visualization, and sensitivity. Understudied IQCs that merit further consideration include out-of-plane resolution, geometric accuracy, uniformity, low-contrast detectability, and co-registration accuracy.

**Conclusions**: Available medical image quality standards provide a blueprint for establishing consensus best practices for photoacoustic image quality assessment and thus hastening PAI technology advancement, translation, and clinical adoption.

## 1 Introduction

Photoacoustic imaging (PAI) is a rapidly emerging modality that has been proposed for numerous clinical applications including cancer detection, mammography, vascular imaging, tissue oximetry, tumor margining, and biopsy/surgical guidance, among others.^1^^–^^5^ This wide range of applications and the novelty of the field has resulted in a large variety in device designs. PAI device performance will generally vary with device design parameters (e.g., transducer geometry, optical source properties) as well as tissue parameters (e.g., properties and morphology). Quantitatively predicting how these parameters influence PAI device performance *in vivo* is challenging. Bench performance test methods can provide insight on design consequences, elucidate device working mechanisms, and help set performance expectations and limitations. Tissue-mimicking phantoms provide an invaluable approach for objective, quantitative evaluation of fundamental image quality characteristics (IQCs) as well as more technology-specific aspects of PAI system performance such as oximetry measurement accuracy, spectral recovery, or chromophore concentration accuracy.^6^^–^^11^

However, no standardized phantom-based performance test methods have been established for PAI. This places a burden on researchers and device developers to design their own phantoms and test methods, thus increasing development time and cost while potentially causing redundancy of efforts across the community. Comparing device test results against those reported in the literature is also challenging given the variation in phantom design and testing methodology. Consensus PAI performance test methods are needed to facilitate consistent and scientifically rigorous, yet least burdensome evaluation of device performance. Such test methods can support many aspects of the medical product life cycle, including device development and optimization, benchmarking or inter-comparison, clinical trial standardization, quality management systems, regulatory evaluation, post-market studies, constancy testing, calibration, and accreditation. The US Food and Drug Administration (FDA) can formally “recognize” voluntary consensus standards as being suitable for regulatory purposes, which can potentially streamline regulatory decision-making.^12^ Standards development is not only a key step in clinical translation and adoption of an imaging modality but may also improve device quality, increase device consistency across manufacturers, and serve as an indicator of technological maturity.

Standardized, phantom-based performance test methods have been developed for mature imaging modalities such as ultrasound, x-ray computed tomography (CT), x-ray mammography, and magnetic resonance imaging (MRI) through standards organizations such as the International Electrotechnical Commission (IEC), International Organization for Standardization (ISO), and National Electrical Manufacturers Association (NEMA). Additionally, consensus documents containing expert recommendations for image quality assessment have been developed by professional societies including the American Association of Physicists in Medicine (AAPM) and the American College of Radiology (ACR), as well as community-led working groups and consortia.^13^^–^^16^ These groups have designed accreditation programs that provide facilities performing medical imaging with recommendations on staff qualifications, equipment characteristics, phantom properties, quality control (QC) routines, and quality assurance (QA) tests. Some phantom manufacturers offer products that are specifically designed to meet the requirements of these standards removing the burden of fabrication and characterization from the developer or end user.^17^^–^^19^ Community interest in addressing these standardization needs is evidenced by the recent establishment of the International Photoacoustics Standardisation Consortium (IPASC), which aims to standardize PAI phantoms and performance test methods.^20^ There is also a similar rise in standards development activities for other biophotonics technologies, including near-infrared cerebral oximeters^21^ and fluorescence-guided surgery.^22^

Our overall goal is to support development of robust, consensus-based performance test methods for emerging PAI devices. We aimed to determine whether available medical imaging standards can be leveraged to inform and guide establishment of standardized test methods for PAI. To this end, we reviewed standards, consensus documents, and clinical accreditation guidelines describing image quality test methods for ultrasound, CT, x-ray mammography, and MRI. We also reviewed the PAI literature to capture the current state of the art in PAI phantom testing, compared findings against available image quality standards for mature modalities, and offered insights and recommendations for future standards development efforts in PAI.

## 2 Image Quality Test Methods for Established Modalities

**Fig. 1:**
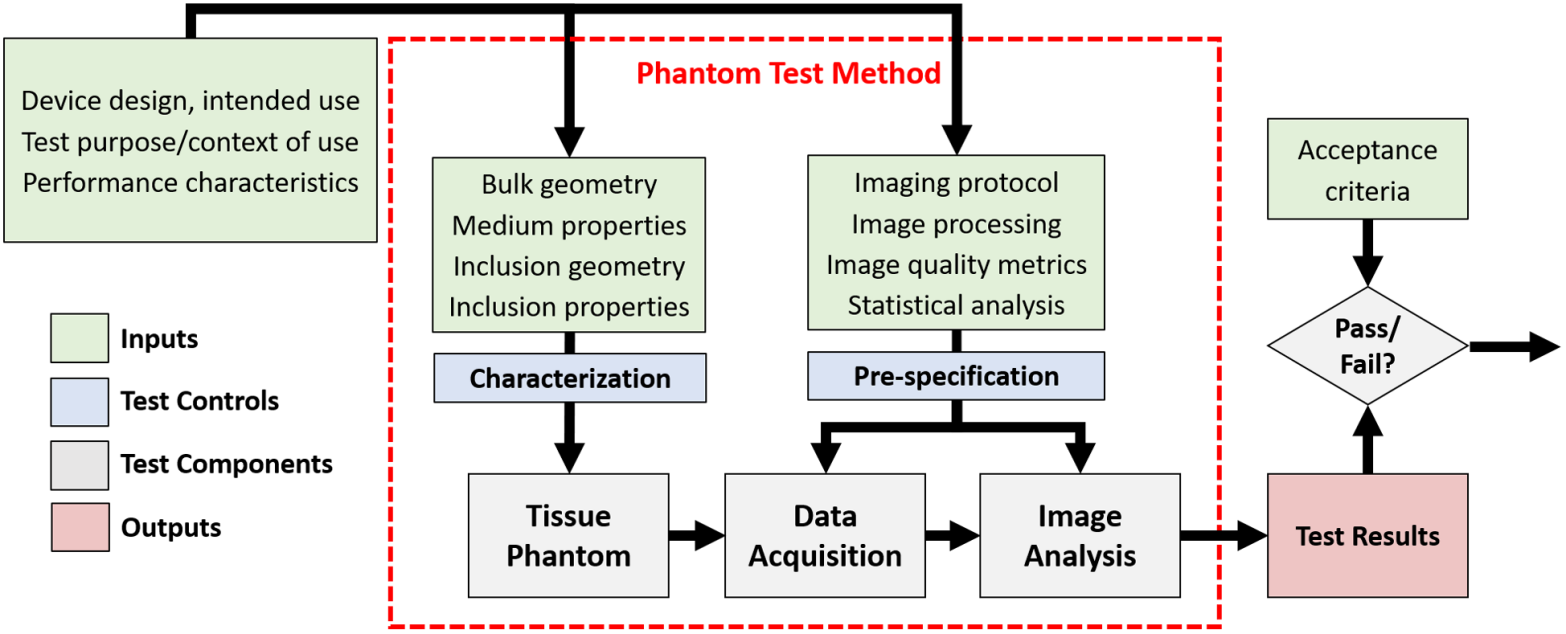
Schematic of a phantom-based image quality test method.

The design of a standardized performance test method should begin with establishing the scope of device types the test applies to, the intended uses of those devices, the purpose of the test, key performance characteristics to be evaluated, and minimum acceptance criteria, if applicable (Fig. 1). Phantom test method specifications include phantom design requirements such as tissue-mimicking material (TMM) properties and the geometry of embedded targets. Phantoms should be rigorously characterized to ensure they meet desired specifications. In addition to phantom design, the methods for data acquisition and analysis also require careful consideration. The test method should provide a detailed protocol for taking phantom measurements, recommend best practices for image processing settings, and define appropriate image quality metrics. The test methodology should be “pre-specified,” meaning that the tester is not permitted to deviate from the specified protocol to produce more favorable outcomes (especially during execution of the test). Protocol modifications may be justifiable in certain situations (novel device configuration and inadequate phantom design), but in those cases the test should be repeated using the modified protocol.

Our review of image quality consensus test methods for ultrasound, CT, x-ray mammography, and MRI included research literature, standards, technical reports, consensus documents, and accreditation program requirements. We found that the scope and content of these documents varied widely. For instance, several clinical QA guidelines specified only high-level testing program requirements such as classes of image quality tests to perform (e.g., a generic requirement to evaluate spatial resolution using an unspecified test method).^23^^–^^27^ These documents also provided requirements for logistics of performance testing such as test report formats, recommended schedules for measurements in constancy testing, and “defect levels” that determine when system repair is needed to restore performance. In this review, we focused on documents that describe specific phantom-based image quality test methods because these fundamental details are of greatest interest for developing consensus test methods for PAI. Our review summarizes standardized test methods for evaluating IQCs most commonly used across all standards and most relevant to PAI including spatial resolution, geometric accuracy, image uniformity, depth of visualization, sensitivity, low-contrast detectability, and artifacts.

### 2.1 Spatial Resolution

**Fig. 2:**
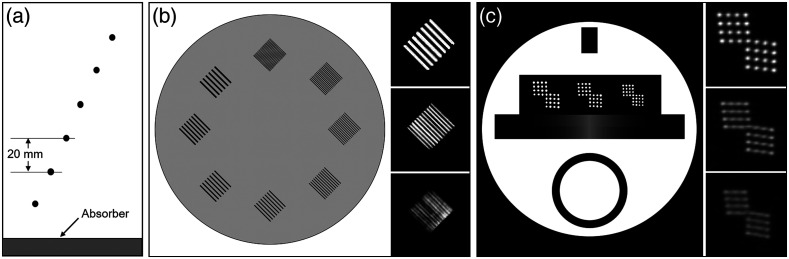
(a) Schematic of an ultrasound PSF wire phantom. (b) Diagram and captured images of a CT resolution phantom containing aluminum bar targets. (c) Illustration and acquired images of MRI resolution phantom containing arrays of water-filled holes. Reproduced and adapted with permission from Refs. 28–30, respectively.

Several standard test methods for evaluating in-plane spatial resolution were available for each of the three modalities, which is not surprising given the well-accepted importance of resolution in medical imaging. A key distinction was whether a test was based on qualitative (subjective) or quantitative (objective) image evaluation. Some ultrasound, CT, and MRI standards used a phantom containing various line or grid patterns with known target spacings [Figs. 2(b) and 2(c)], and resolution was determined as the spacing of the finest target in which the reader can distinguish the line pattern.^14^^,^^15^^,^^28^^,^^29^^,^^31^^,^^32^ However, this approach is subjective, depending on the individual reader. Other standards describe objective, quantitative resolution tests, for instance by measuring the width of the point spread function (PSF) or line spread function (LSF) of a single sub-resolution target, usually specified as the full width at half maximum (FWHM), or less often, at tenth maximum (FWTM) [Fig. 2(a)].^14^^,^^30^ Placing several targets at various locations in the field-of-view also allows characterization of spatial variation in resolution. Another more comprehensive approach is to measure the modulation transfer function (MTF), a well-known approach used in optical imaging and endoscopy standards.^33^^,^^34^ A CT standard described computing MTF as the normalized Fourier transform of the PSF or LSF produced by a small, high-contrast wire, bead, or edge target embedded in a minimally attenuating background material. Spatial resolution was evaluated by reporting both the 10% and 50% points on the MTF curve.^35^ It is worth noting that the common approach of measuring contrast, $C=(I_{\max}-I_{\min})/(I_{\max}+I_{\min})$, versus spatial frequency in square-wave or bar patterns, such as the well-known 1951 USAF target, yields the contrast transfer function (CTF), which is not equal to the MTF.^34^

Most resolution tests recommended use of high contrast targets at pre-specified positions. One ultrasound standard recommended using either (1) moderate-contrast nylon filaments in a “working liquid” with speed of sound 1540±15  m/s, low acoustic attenuation (<0.1  dB/cm/MHz), and negligible scattering; or (2) high-contrast metal wires in a TMM with the same speed of sound, bio-relevant attenuation (0.5±0.05  dB/cm/MHz), and an unspecified “moderate” level of scattering.^30^ The first approach represents an engineering test under ideal conditions that may be useful for basic system characterization, and the latter represents a test closer to real-world conditions that may better predict *in vivo* performance. Accreditation programs often prescribed well-established, commercially available phantoms, some of which contained several “modules” for testing different IQCs.^32^^,^^36^ For example, the ACR CT phantom has an in-plane resolution module containing eight aluminum bar patterns ranging from 4 to 12 line pairs per centimeter embedded in a biologically relevant background [Fig. 2(b)]. The ACR MRI phantom contains a resolution module consisting of water-filled cylindrical cavities in various grid patterns [Fig. 2(c)].^32^^,^^36^

**Fig. 3:**
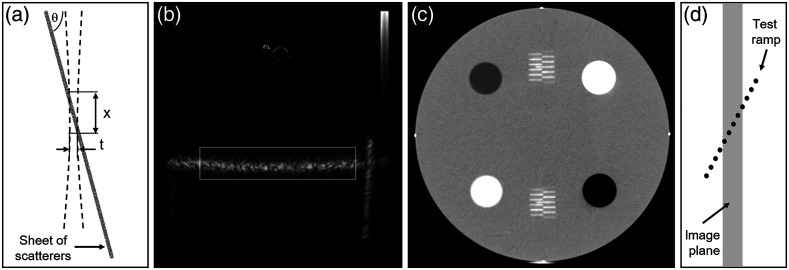
(a) and (b) Diagram and captured image of an ultrasound slice thickness phantom using an angled plane (θ=75  deg) of scatterers, showing a typical ultrasound beam [dashed lines in (a)]. Reproduced and adapted with permission from Refs. 30 and 38, respectively. (c) and (d) Diagram of a CT slice thickness phantom using filament ramps. Reproduced and adapted with permission from Ref. 28.

Standards also specified tests for evaluating elevational (out-of-plane) resolution or section/slice thickness. These test methods typically used an angled object of known properties and dimensions slanted relative to the imaging plane.^14^^,^^15^^,^^28^^–^^30^^,^^32^^,^^35^^,^^37^ For example, an ultrasound test method describes scanning the transducer across a hyperechoic slab, angled at 75 deg relative to phantom surface, which appears in cross-sectional images as a rectangular object at variable depth [Figs. 3(a), 3(b)].^30^ Elevational resolution, t, was determined as t=x/tan(75  deg), where x is the vertical height of the object. The ACR CT phantom contained two ramps of short wires positioned along out-of-plane inclines in opposite directions with elevational wire spacing of 0.5 mm [Figs. 3(c), 3(d)].^28^ Slice thickness was computed as half the number of wires appearing at least 50% as bright as the central wires. MRI slice thickness has been determined by measuring FWHM of the signal intensity profile produced by a thin slab inclined at a 5 deg to 12 deg angle embedded in an MR-inactive material.^37^ Slice thickness was calculated as the product of the FWHM of the trapezoidal profile and tan(α). An alternative to imaging angled targets is to scan a small point or line target in the elevational direction. For instance, an ultrasound standard described elevational resolution measurement by scanning a vertically oriented wire in a water bath,^30^ whereas a CT standard characterized slice thickness by scanning a <0.1  mm-thick disk or bead.^35^ Goodsitt et al.^14^ described a “less frequent” ultrasound test based on scanning an anechoic spherical object, although no explicit method for quantifying elevational resolution was provided.

### 2.2 Geometric Accuracy

**Fig. 4:**
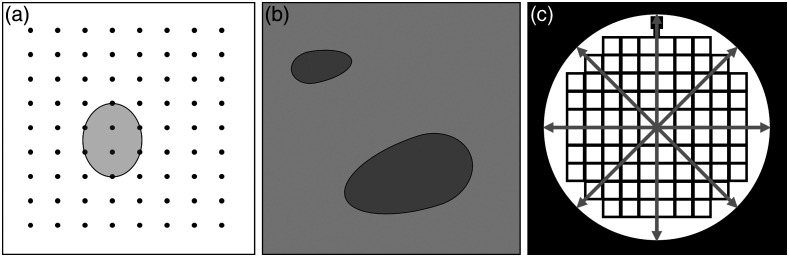
Illustrations of (a) filament array for 1D distance and 2D area measurement accuracy (e.g., area of the drawn ellipse), and (b) ovoid inclusion phantoms for 1D, 2D, and 3D ultrasound spatial measurement accuracy testing (b). Reproduced and adapted with permission from Ref. 30. (c) Grid pattern phantom for MRI geometric accuracy evaluation. Reproduced and adapted with permission from Ref. 32.

Geometric accuracy, the ability of an imaging system to accurately represent tissue morphology, can be characterized by spatial measurement accuracy and image distortion. Assessment of tissue structure and geometry commonly involves the use of software-based image caliper tools in 1D (e.g., tissue layer thickness, distance between objects), 2D (e.g., vessel cross-sectional area), or 3D (e.g., tumor volume). In-plane spatial measurement accuracy test methods were available for ultrasound, CT, and MRI.^14^^,^^15^^,^^28^^–^^30^^,^^32^ These methods recommended imaging phantoms containing an array of high contrast targets [Fig. 4(a)] or a grid pattern [Fig. 4(c)] and comparing measured target distances in the image to known target distances. This approach can be used for linear, curvilinear, and circumferential measurements. Similarly, the accuracy of computed 2D cross-sectional areas and 3D inclusion volume can be evaluated by imaging a phantom containing 3D ovoid inclusions [Fig. 4(b)].^30^

Image distortion denotes spatial variation in magnification, such as well-known barrel or pincushion distortion effects in optical imaging. Distortion can also be asymmetric; for instance, incorrect ultrasound image reconstruction (e.g., poor speed of sound parameter) can cause significant distortion in the axial direction. An ultrasound consensus document described a qualitative distortion test by imaging a spherical or cylindrical phantom inclusion, which will appear as flattened or extended ovals if the image is distorted.^14^ Quantitative distortion tests often leveraged the same target grid phantoms used spatial resolution testing. One MRI distortion test recommended using a phantom containing a uniform grid or hole pattern to compute coefficient of variation of adjacent grid target spacings.^36^ A different MRI approach involved imaging a phantom of known dimensions in all three orthogonal planes and computing the percent of geometric distortion (%GD) in each plane as

$$\%\mathrm{GD}=100\times \frac{\Delta_{\text{actual}}-\Delta_{\text{measured}}}{\Delta_{\text{measured}.}}.$$ (1)

where $\Delta_{\text{actual}}$ is the actual phantom dimension and $\Delta_{\text{measured}}$ is the dimension as measured on the image.^36^

### 2.3 Uniformity and Depth of Visualization

**Fig. 5:**
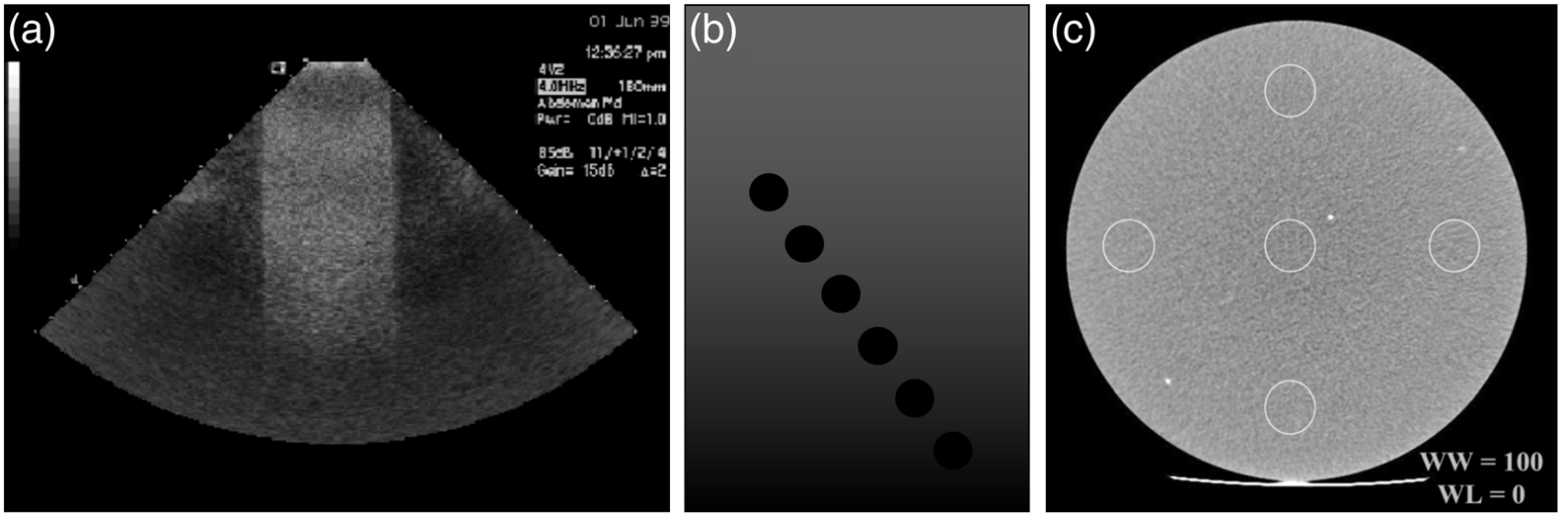
(a) Ultrasound image of homogeneous phantom for evaluating depth of visualization. (b) Diagram for an ultrasound depth phantom containing anechoic inclusions in homogeneous background. (c) Captured image of the ACR CT uniformity phantom, showing circular ROIs. Reproduced and adapted with permission from Refs. 14, 28, and 39, respectively.

Image uniformity describes spatial variation in sensitivity across an image field. Several documents recommended imaging a homogeneous, biologically relevant phantom and drawing several circular regions of interest (ROIs) to measure variations in image intensity across the field-of-view.^15^^,^^28^^,^^29^^,^^31^^,^^32^^,^^35^^,^^37^ In an ACR CT accreditation program, the mean CT number was computed for ROIs at the center and four edge positions [Fig. 5(c)], and uniformity was quantified as the absolute error between each edge ROI mean and the center ROI mean.^28^ Similarly, an ACR MRI consensus document recommended drawing two small ROIs over regions having highest and lowest signal based on qualitative inspection.^32^ Mean signal intensity in these two ROIs (${\mathrm{ROI}}_{\text{high}}$, ${\mathrm{ROI}}_{\text{low}}$) was measured to compute percent integral uniformity (PIU) as

$$\mathrm{PIU}=100\%\times [1-\{\frac{{\mathrm{ROI}}_{\text{high}}-{\mathrm{ROI}}_{\text{low}}}{{\mathrm{ROI}}_{\text{high}}+{\mathrm{ROI}}_{\text{low}}}\}].$$ (2)

While CT and MRI systems can typically visualize signals within the entire field-of-view, ultrasound systems have finite imaging depth due to tissue attenuation and limited viewing angle. Thus, ultrasound documents considered depth of visualization or maximum penetration depth, the maximum depth to which the system provides useful information, but neglected lateral image uniformity.^14^^,^^38^^–^^40^ Maximum imaging depth was often assessed by imaging phantoms containing arrays of cylindrical inclusions that are anechoic or have specified contrast positioned at different depths [Fig. 5(b)], identifying the deepest visible inclusion by inspection or the depth at which background texture “can barely be seen reliably.”^14^ A more quantitative approach computed the signal-to-noise ratio (SNR) of anechoic inclusions at various depths as

$$\mathrm{SNR}=\frac{\left(m_{\text{target}}-m_{\text{background}}\right)}{\sigma_{\text{background}}},$$ (3)

where $m_{\text{target}}$ is the mean target ROI intensity, $m_{\text{background}}$ is the mean background ROI intensity, and $\sigma_{\text{background}}$ is the standard deviation of the background ROI.^14^ However, since SNR values are only available at discrete depths where targets are placed, test results may depend on phantom design. Another standard described the use of a large, homogeneous phantom with specified acoustic attenuation and backscatter coefficient over 1 to 15 MHz [Fig. 5(a)].^39^ Images were acquired in the phantom as well as with the transducer in air to measure electronic noise, and the maximum depth of penetration was defined as the axial location where the phantom signal decays to 1.4 times the noise signal, which corresponds to an SNR of 1 using the following definition:

$$\mathrm{SNR}(j)=\sqrt{\frac{A{\left(j\right)}^{2}}{A^{\prime }{\left(j\right)}^{2}}-1.}$$ (4)

where A(j) is the mean gray level of all pixels at a given depth, j, and $A^{\prime }(j)$ is a similar measurement in the noise image.^39^

### 2.4 Sensitivity and Low-Contrast Detectability

**Fig. 6:**
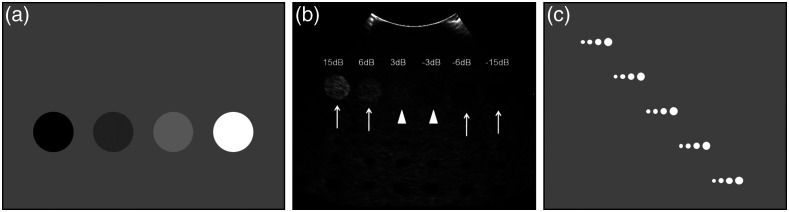
(a) Diagram and (b) acquired image of an ultrasound phantom for local dynamic range measurements. (c) Diagram of an ultrasound low-contrast detectability phantom. Reproduced and adapted with permission from Refs. 39, 43, and 44, respectively.

Sensitivity was most often used to describe the detection limit of an imaging system,^14^^,^^39^ but it may also describe the rate of change in image signal intensity versus target properties (e.g., target radioactivity, chromophore concentration).^41^^,^^42^ An ultrasound standard defined a closely related IQC, local dynamic range, as the difference in dB of echo amplitudes that produce minimum and maximum gray levels. Local dynamic range was evaluated using a phantom incorporating inclusions with different levels of relative contrast (e.g., −6  dB, −3  dB, +3  dB, and +6  dB) placed at the same depth within a biologically relevant echogenic background [Figs. 6(a) and 6(b)]. Local dynamic range was determined by finding the intercepts at 0 and 255 gray levels for a linear regression of ROI-averaged target amplitude versus known target contrast.^39^ This standard also requires image processing settings to be reported for any local dynamic range measurement, as these controls will alter test results.

Low-contrast detectability denotes the ability to distinguish objects with similar brightness to the image background. Target size is typically varied in such tests to enable contrast-detail analysis, the combined evaluation of how object contrast and size impact object detectability. An ultrasound standard described an echogenic phantom containing arrays of 1- to 2-mm diameter anechoic spherical inclusions at various depths, where the smallest inclusion per depth was determined by inspection [Fig. 6(c)].^43^ An alternative ultrasound approach used a phantom containing 10  cm×20  cm conical inclusions with different contrast levels.^43^ The transducer was scanned along the cone axis to change the in-plane cross-sectional area of the target cones, and the minimum detectable size for each contrast level was determined qualitatively.

**Fig. 7:**
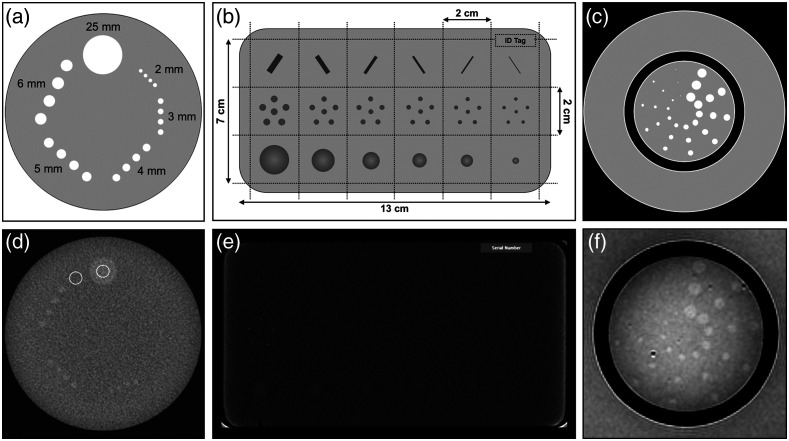
(a) Diagram and (d) acquired image of a CT low-contrast detectability phantom. Reproduced and adapted with permissions from Refs. 28 and 31, respectively. (b) Diagram and (e) acquired image of the ACR Digital Mammography phantom. Reproduced and adapted with permission from Ref. 45. (c) Diagram and (f) acquired image of the ACR MRI low-contrast detectability spoke phantom. Reproduced and adapted with permission from Ref. 32.

Test methods for CT system low-contrast detectability involved a phantom containing arrays of cylindrical inclusions (2 to 10 mm in diameter) embedded in a biologically relevant medium [Figs. 7(a) and 7(d)].^15^^,^^28^^,^^31^^,^^35^ Detectability was either determined qualitatively by identifying the smallest set of “clearly delineated” inclusions or quantitatively by computing contrast-to-noise ratio (CNR):

$$\mathrm{CNR}=\frac{m_{\text{target}}-m_{\text{background}}}{\sigma_{\text{background}}},$$ (5)

where $m_{\text{target}}$ is the mean signal of a target ROI, and $m_{\text{background}}$ and $\sigma_{\text{background}}$ are the mean signal and standard deviation of a local inclusion-specific background ROI.^31^ A similar MRI phantom contained radial “spokes” of 1.5- to 7-mm diameter cylindrical inclusions [Figs. 7(c) and 7(f)], as well as several elevational slices with inclusions at different contrast levels.^32^^,^^36^ Low-contrast detectability was determined as the number of spokes for which all three targets are distinguishable for each contrast level.

The ACR x-ray mammography QC manual prescribed an approach to evaluate low-contrast detectability using an approved ACR digital mammography phantom.^45^ The phantom simulated a compressed breast of average density and contained a wax insert with groups of biomimetic inclusions relevant to breast cancer findings, such as tissue fibers (0.3 to 0.89 mm), specks representing calcifications (0.14 to 0.33 mm), and tumor-mimicking masses (0.2 to 1.0 mm) [Figs. 7(b) and 7(e)]. Minimum performance criteria were specified in terms of the smallest targets detected by a trained reader such as a radiologist. This approach differs significantly from other low-contrast detectability phantoms in that it uses three types of semi-idealized biological target features, as opposed to a more objective/quantitative but generalized evaluation using a single inclusion geometry. Both paradigms have merits and may be useful in device characterization and QC settings.

### 2.5 Artifacts

**Fig. 8:**
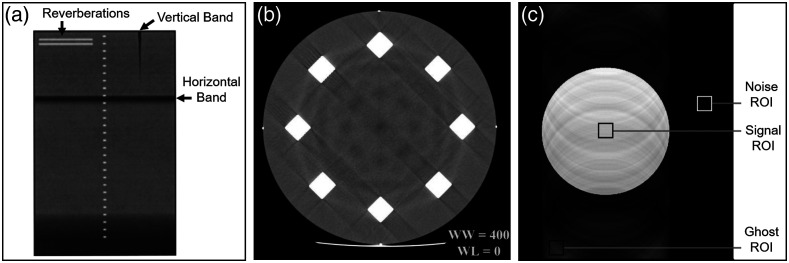
(a) Phantom-based evaluation of ultrasound artifacts; (b) CT streak artifacts; and (c) MRI ghost artifacts. Reproduced and adapted with permission from Refs. 14, 28, and 37 respectively.

An image artifact is a visualized feature that is misrepresentative of the true object morphology and cannot be explained by random noise.^37^ Artifactual shapes can either be reproductions of existing structures in the imaged object (e.g., ghosts, faint copies of an object superimposed on the image and displaced from its original location) or shapes unrelated to the imaged object. Artifacts can obscure true features of clinical interest, adversely affect diagnostic image interpretation, and corrupt phantom measurements of other performance characteristics. Test methods for artifacts tended to be less quantitative than those for other performance characteristics. AAPM QC procedures included evaluation of ultrasound image artifacts in a homogeneous tissue-mimicking phantom.^14^ Phantom images are inspected for streak artifacts not caused by beam coupling or phantom imperfections [Fig. 8(a)], and any deviations from the expected uniform image that rise to an action level (at which system repair should be made) or defect level (at which performance becomes severely affected) above the background are to be addressed. In the ACR CT accreditation program, artifact assessment relies on visual inspection of phantom images and manufacturer-specific corrective actions [Fig. 5(c)].^15^^,^^23^^,^^28^^,^^31^ These documents provided example images illustrating cupping, helical, ring, and streak/line artifacts [Fig. 8(b)]. MRI ghost artifacts, which are typically caused by patient motion or vibration and can be significant in low-contrast scenarios, can be evaluated using a homogeneous phantom as used for uniformity testing.^32^ A large primary ROI was drawn over the phantom as well as several background ROIs outside of the phantom, from which the ghosting ratio computed as

$$R_{\text{ghosting}}=|\frac{\left[S_{\text{top}}+S_{\text{bottom}}]-[S_{\text{left}}+S_{\text{right}}\right]}{2\times S_{\text{large}}}|,$$ (6)

where S is the average pixel intensity in each ROI. A similar approach described in IEC 62464-1:2018 uses ROI measurements in a homogeneous phantom to compute ghost-to-signal ratio [Fig. 8(c)], ghost-to-noise ratio, and SNR:

$$\mathrm{GSR}=\frac{I_{G}}{S},\;\mathrm{GNR}=\frac{I_{G}}{I_{N}},\;\mathrm{SNR}=\frac{S}{I_{N}},$$ (7)

where $I_{G}$ is the mean ghost ROI signal, S is mean phantom ROI signal, and $I_{N}=\sigma /0.655$ is the standard deviation of the background ROI, σ, corrected for image reconstruction effects.^37^ The standard required reporting of all three metrics.

## 3 Current Image Quality Evaluation Practices in Photoacoustic Imaging

We used Web of Science to search for peer-reviewed journal articles published from 2010 to 2020 on PAI phantoms. This yielded 686 articles (search terms: [photoacoustic OR optoacoustic] AND imaging AND phantom). However, there was considerable variation in reported phantom complexity, characterization, and context of use. To better align with our review of medical imaging standards, we excluded articles that (1) tested photoacoustic microscopy, elastography, non-imaging spectroscopy, flowmetry, or 1D depth profiling systems; (2) only used digital/numerical phantoms or *ex vivo* tissue; and (3) focused on non-image quality performance aspects such as oximetry measurement accuracy, fluence correction, or quantitative imaging. We focused our review on the 119 of the remaining 308 articles that used phantoms to quantify one or more specific IQCs, rather than only describing TMM development or qualitative performance testing. These articles included phantom studies of both 2D and 3D PAI systems.

A wide variety of background phantom materials was observed, including water,^46^^,^^47^ Intralipid,^48^^–^^50^ and various TMMs such as hydrogels (agar, gelatin, and polyvinyl alcohol),^51^^–^^56^ polyurethane,^57^^–^^59^ silicone,^60^ gel wax,^8^ styrene-ethylene/butylene-styrene polymer,^61^ polydimethylsiloxane,^62^^,^^63^ and polyvinyl chloride plastisol (PVCP).^48^^,^^64^^,^^65^ Of the 119 studies of interest, 64 (54%) performed testing on targets immersed in non-turbid water baths or gels, rather than embedded in tissue-mimicking phantoms. This approach may be suitable in some cases to determine ideal performance (e.g., resolution testing) but may not be appropriate for IQCs that vary significantly with tissue attenuation (e.g., imaging depth). Only 36 (65%) and 8 (15%) of 55 studies using turbid phantoms characterized phantom optical and acoustic properties, respectively. In some cases, expected TMM properties were reported from previous literature, but many studies provided no discussion of phantom properties nor justification of their biological relevance. Phantom properties should be well-characterized to demonstrate biological relevance for an intended imaging application.

**Fig. 9:**
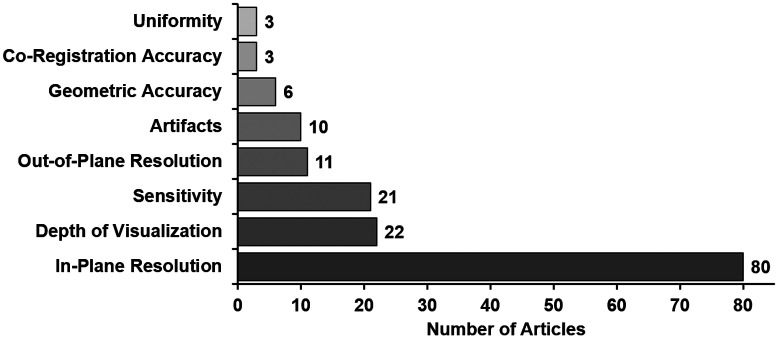
Most commonly tested IQCs in reviewed PAI articles (some articles evaluated multiple IQCs).

In-plane spatial resolution was by far the most commonly tested IQC, followed by depth of visualization and sensitivity (Fig. 9); other IQCs frequently encountered in medical imaging standards were significantly understudied. This may have been due to prioritization of IQCs that demonstrate the proposed advantages of PAI, namely, high-resolution imaging to detect deep, absorptive targets.^66^ We also observed high variation in how IQCs were quantified, particularly for metrics related to target contrast and detectability. Reported image quality metrics included photoacoustic signal intensity (arbitrary units), SNR, signal-to-background ratio (SBR), contrast, contrast ratio (CR), and CNR. Adding to the confusion, these metrics have been defined many different ways (Table 1) or occasionally not explicitly defined. Note that the ratio of mean target image amplitude to mean background image amplitude (S/B) has been called SNR, SBR, CR, and CNR! The term SNR also requires careful interpretation as in some cases it referred to quality of raw, un-beamformed photoacoustic signals. To avoid ambiguity, image quality metrics and methods for their calculation should always be explicitly defined in a performance test method. It is important that both target contrast and background variation be considered when evaluating object detectability. One self-consistent set of metric definitions capturing both of these effects that we have employed is $\mathrm{SNR}=S/\sigma_{B}$, CR=SBR=S/B, and $\mathrm{CNR}=(S-B)/\sigma_{B}$, which also yields the relationship CNR=SNR(1−1/SBR).^92^ One benefit of developing consensus documents is the establishment of standardized terms and definitions to enable reproducible data analysis and comparison of test results between systems.

**Table 1:** Reported definitions of image quality metrics in PAI studies, ranked in order of our descending preference (parentheses). S = mean target amplitude or power, B = mean background amplitude or power, $\sigma_{B}$ = background standard deviation, “RMS” denotes root-mean-square, “max” and “min” denotes maximum and minimum values, subscript “2” denotes analysis of two-frame subtracted image, “pre-log” denotes using pre-log compression image amplitudes, and ‘global’ denotes analysis of the entire image (not ROIs, as for other definitions here).

IQ metric	Reported definitions		
SNR	(1) $\frac{S}{\sigma_{B}}$^67^^–^^71^	(6) $\frac{S}{B}$ ^53^	(11) $\frac{\;S_{2}}{\sigma_{B,2}}\sqrt{2}$ ^73^
	(2) $\frac{S_{\mathrm{pre}-\log}\;}{\sigma_{B,\mathrm{pre}-\log}}$^48^	(7) $20\log_{10}\frac{S}{B}$ ^72^^,^^73^	(12) $\frac{S_{\max}}{B_{\max}}$ ^74^
	(3) $10\;\log_{10}\;\frac{S}{\sigma_{B}}$^75^	(8) $10\log_{10}\frac{S}{B}$ ^46^^,^^76^	(13) $\frac{S_{\max}}{B_{\mathrm{RMS}}}$ ^77^
	(4) $\frac{S_{\max}}{\sigma_{B}}$ ^55^	(9) $20\log_{10}\frac{S_{\max}-S_{\min}}{\sigma_{S}}$^78^	(14) $\frac{S-B}{\sqrt{\sigma_{S}^{2}+\sigma_{B}^{2}}}$^62^
	(5) $20\;\log_{10}\;\frac{S_{\max}}{\sigma_{B}}$ ^79^^,^^80^	(10) $20\;\log_{10}\;\frac{S_{\mathrm{RMS}}}{B_{\mathrm{RMS}}}$ ^81^	(15) $10\;\log_{10}\;\frac{S-B}{\sigma_{B}}$ ^82^
SBR	(1) $\frac{S}{B}$ ^57^^,^^83^	(2) $\frac{{\left(S_{\max}\right)}^{2}}{B^{2}}$ ^84^	
Contrast or CR	(1) $\frac{S}{B}$ ^85^	(3) $20\log_{10}\frac{S_{\mathrm{pre}-\log}}{B_{\mathrm{pre}-\log}}$ ^80^	(5) $\frac{S-B}{S+B}$ ^86^
	(2) $20\;\log_{10}\;\frac{S}{B}$ ^68^^,^^71^	(4) $\frac{S-B}{B}$ ^62^^,^^87^	
CNR	(1) $\frac{S-B}{\sigma_{B}}$ ^48^	(5) $\frac{S-B}{\sqrt{\sigma_{S}^{2}+\sigma_{B}^{2}}}$^69^	(9) $20\;\log_{10}\;\frac{S}{\sigma_{B}}$^88^
	(2) $20\;\log_{10}\;\frac{S-B}{\sigma_{B}}$ ^73^	(6) $\frac{\left|S-B\right|}{\sqrt{\sigma_{S}^{2}+\sigma_{B}^{2}}}$^71^^,^^79^	(10) $10\;\log_{10}\;\frac{S_{\mathrm{RMS}}-\sigma_{B}}{\sigma_{B}}$ ^89^
	(3) $\frac{\left|S-B\right|}{\sigma_{B}}$ ^68^	(7) $\frac{S_{\mathrm{global}}-B_{\mathrm{global}}}{\sigma_{B,\text{global}}}$ ^90^	(11) $\frac{S}{B}$ ^58^
	(4) $20\;\log_{10}\;\frac{\left|S-B\right|}{\sigma_{B}}$ ^72^^,^^91^	(8) $\frac{S}{\sigma_{B}}$^90^	

### 3.1 Spatial Resolution

The most common approach for evaluating in-plane spatial resolution was to measure axial and/or lateral dimensions of the LSF produced by one or more line targets perpendicular to the image plane. It is worth noting that unlike some modalities described in Sec. 2, in-plane resolution is often anisotropic in PAI. This approach is essentially identical to resolution test methods described in ultrasound standards.^30^ The ideal PAI resolution target should be much smaller than the resolution limit and produce high image contrast. Target size varied widely (6  μm to 1 mm) due to the broad range of minimum size requirements for PAI devices with different resolution limits. Line target materials included metal wires or filaments (tungsten, steel, copper, aluminum, or unspecified metal),^48^^,^^60^^,^^67^^,^^78^^,^^79^^,^^93^^–^^99^ carbon fibers,^100^^–^^103^ threads,^51^^,^^104^^,^^105^ sutures,^48^^,^^89^^,^^106^^–^^108^ graphite rods (pencil lead),^50^^,^^109^^,^^110^ or human/horse hairs.^10^^,^^86^^,^^111^^–^^118^ Some studies imaged inkjet-printed target patterns on paper or transparency film suspended in water or a tissue-mimicking medium.^90^^,^^119^ Almost all studies computed resolution as the FWHM (−6  dB width) of the measured PSF or LSF, although other metrics were observed including −3  dB width^60^^,^^78^ or half the FWTM.^110^ While targets were often aligned perpendicular to the image plane, some photoacoustic CT studies used line targets parallel to the plane.^10^^,^^114^ An alternative approach was to image spherical point targets such as 10- to 200-μm black polyethylene microspheres,^11^^,^^51^^,^^55^^,^^56^^,^^119^^–^^123^ 100- to 200-μm graphite particles,^124^^,^^125^ or 50-μm polyamide particles.^126^ A few papers evaluated resolution using pairs of adjacent targets such as crossed threads, for instance using Sparrow’s resolution criterion.^51^ This method yielded somewhat larger results versus 50-μm microspheres (189  μm versus 129±16  μm), which was attributed to out-of-plane absorber contributions. Another alternative approach for lateral resolution was to scan a 1951 United States Air Force (USAF) target immersed in water^127^ or beneath a solid phantom^88^^,^^128^ and measure bar FWHM or contrast. However, it may be more appropriate to measure resolution with this target by computing the CTF or reporting line pairs per mm of the smallest discernable pattern by inspection. Also, this method requires vertical transducer scanning or different phantom layer thicknesses to characterize variations in resolution versus depth, whereas filament grids readily provide this information.

**Fig. 10:**
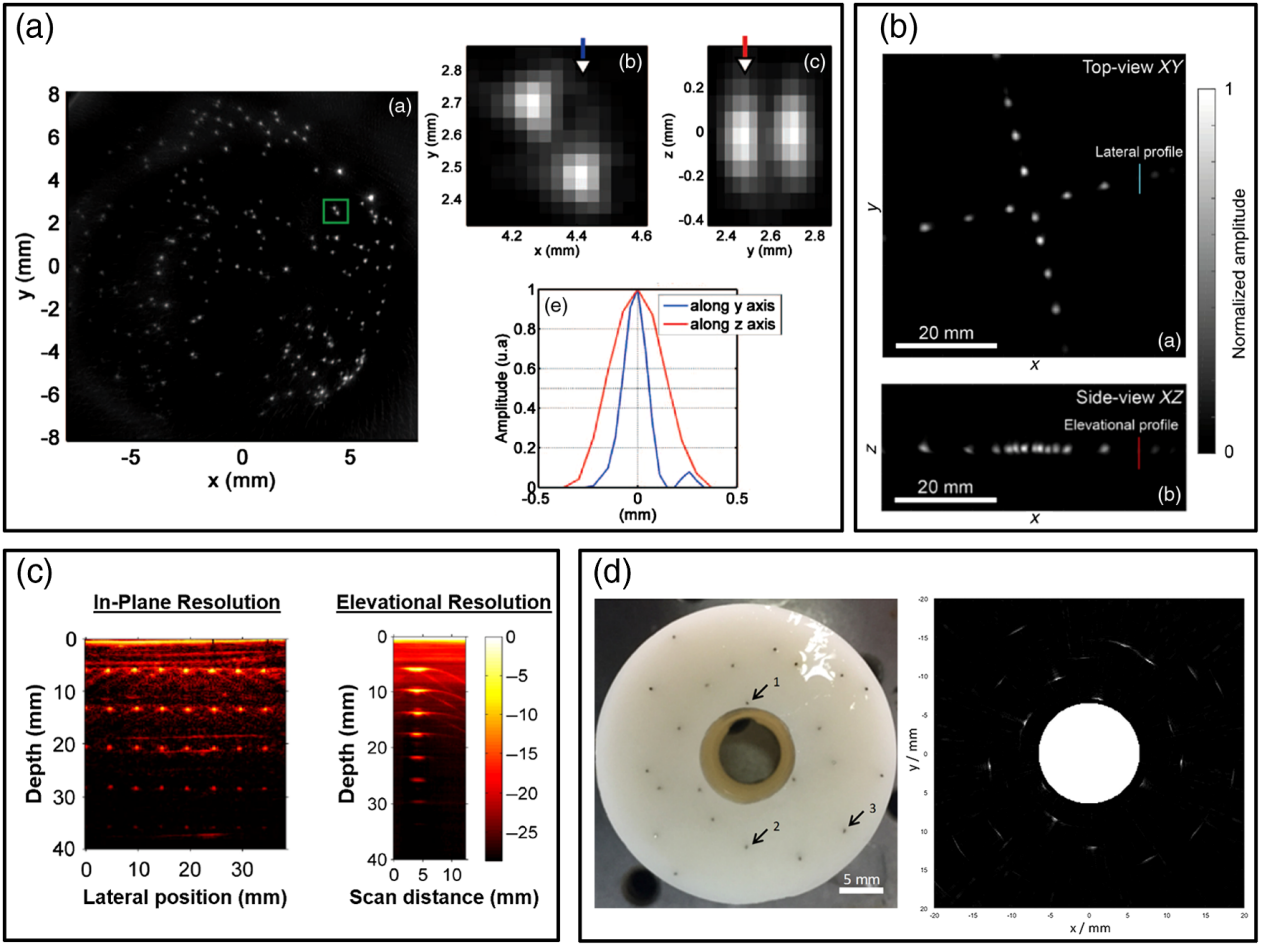
Representative approaches to evaluate photoacoustic image in-plane and elevational resolution, including (a) black polyethylene spheres in agar; (b) black epoxy droplets in water; (c) steel wires in PVCP; or (d) metal wires in agar. Reproduced and adapted with permission from Refs. 46, 48, 51, and 99, respectively.

Unlike in-plane spatial resolution, elevational or out-of-plane resolution was less frequently considered. Medical imaging standards (Sec. 2) often used angled targets for elevational resolution testing, but these methods may not be acceptable for PAI due to light diffusion and limited elevational optical focusing. However, elevational resolution can often be measured using in-plane resolution phantoms—a concept that was seen in image quality standards (Sec. 2.1) (Fig. 10). We previously demonstrated this by scanning a column of steel wires in Intralipid or PVCP phantoms along the elevational direction to measure elevational FWHM versus target depth.^48^ In addition to wire targets, spherical absorbers such as 50- to 100-μm black microspheres^11^^,^^51^^,^^122^^,^^129^ or 0.5- to 1.5-mm black epoxy drops,^46^ have also been used for both in-plane and elevational resolution as the targets are sufficiently small in three dimensions. Another approach suited to photoacoustic CT was to measure the edge spread function of a small needle lowered into the image plane.^79^

Resolution target size varied from ∼1 to 10 times smaller than the measured FWHM, and it is unclear what size requirements are needed to ensure accurate resolution measurements. An MRI slice thickness test recommended feature size at least five times smaller than the FWHM,^37^ whereas an ultrasound resolution test defined sub-resolution line targets such that a ten-fold reduction in diameter would not change apparent target size.^30^ FWHM measurements should be interpreted carefully; if the FWHM is close to the actual target size, the target may not be sufficiently smaller than the resolution limit. PAI resolution should be assessed by measuring FWHM of high contrast, sub-resolution line or point targets positions placed at known locations throughout the field-of-view.

### 3.2 Geometric Accuracy, Distortion, and Co-Registration Accuracy

While geometric accuracy was a common consideration in standardized medical imaging test methods, few PAI articles reported specific geometric accuracy test methods (Fig. 9). Two of our group’s studies leveraged spatial resolution phantoms for this purpose, in similar fashion to standardized test methods (Sec. 2.2). In one study, vertical and horizontal distances between steel filaments in a rectangular grid pattern in a turbid PVCP phantom were measured based on location of the brightest pixel.^48^ These values were compared to nominal target spacing as well as distances measured in co-registered ultrasound images. Another study used a two-layer PVCP phantom with an irregular boundary representing breast fat-glandular tissue interfaces to study the impact of heterogeneity on axial position error of embedded steel wire targets.^130^ Another study used a stacked-layer phantom to evaluate accuracy of PAI-measured layer thickness measurements for skin burn assessment.^131^ This phantom was comprised of thin inkjet-printed patterned polymer sheets containing red dye placed between slabs of turbid acrylic polymer. Similarly, one study evaluated accuracy of target localization (depth) measurements versus target blood content and size using turbid agarose phantoms containing blood-filled spherical gel lesions.^132^ PAI distortion was rarely tested or quantified, although it is well known that improper reconstruction parameters such as speed of sound can distort images, especially in the vertical direction. One study evaluated distortion by imaging a square loop target embedded in a brain-mimicking gelatin phantom beneath *ex vivo* ovine skull.^133^ Distortion due to poor image acquisition settings may be corrected or calibrated, but tissue effects cannot always be avoided or completely mitigated. Especially in the latter scenario, distortion should be included in photoacoustic image quality testing. While no specific distortion test method was described in the literature, a filament grid phantom embedded in a phantom with well-characterized acoustic properties (Sec. 3.1) may be a reasonable approach.

Due to the nature of PAI technology, many PAI systems allow the collection of co-registered photoacoustic and ultrasound images. As with geometric accuracy, US-PAI co-registration accuracy is often not explicitly characterized but can be evaluated using spatial resolution phantoms to compare apparent target positions between US and PA images using either qualitative^134^ or quantitative approaches.^48^^,^^108^ MRI-PAI co-registration has been calibrated using fiducial markers comprised of channels filled with gold nanoparticles and gadolinium solution in an Intralipid-agar phantom.^135^ Additionally, one study characterized localization accuracy of tissue surface-generated photoacoustic signals as fiducial markers for co-registering ultrasound images and stereo camera video.^136^ Co-registration was generally quantified using maximum or average target registration error (TRE), the Euclidean distance between matched points in different images. Co-registration accuracy should be tested in applications combining PAI with other imaging modalities.

### 3.3 Depth of Visualization and Uniformity

**Fig. 11:**
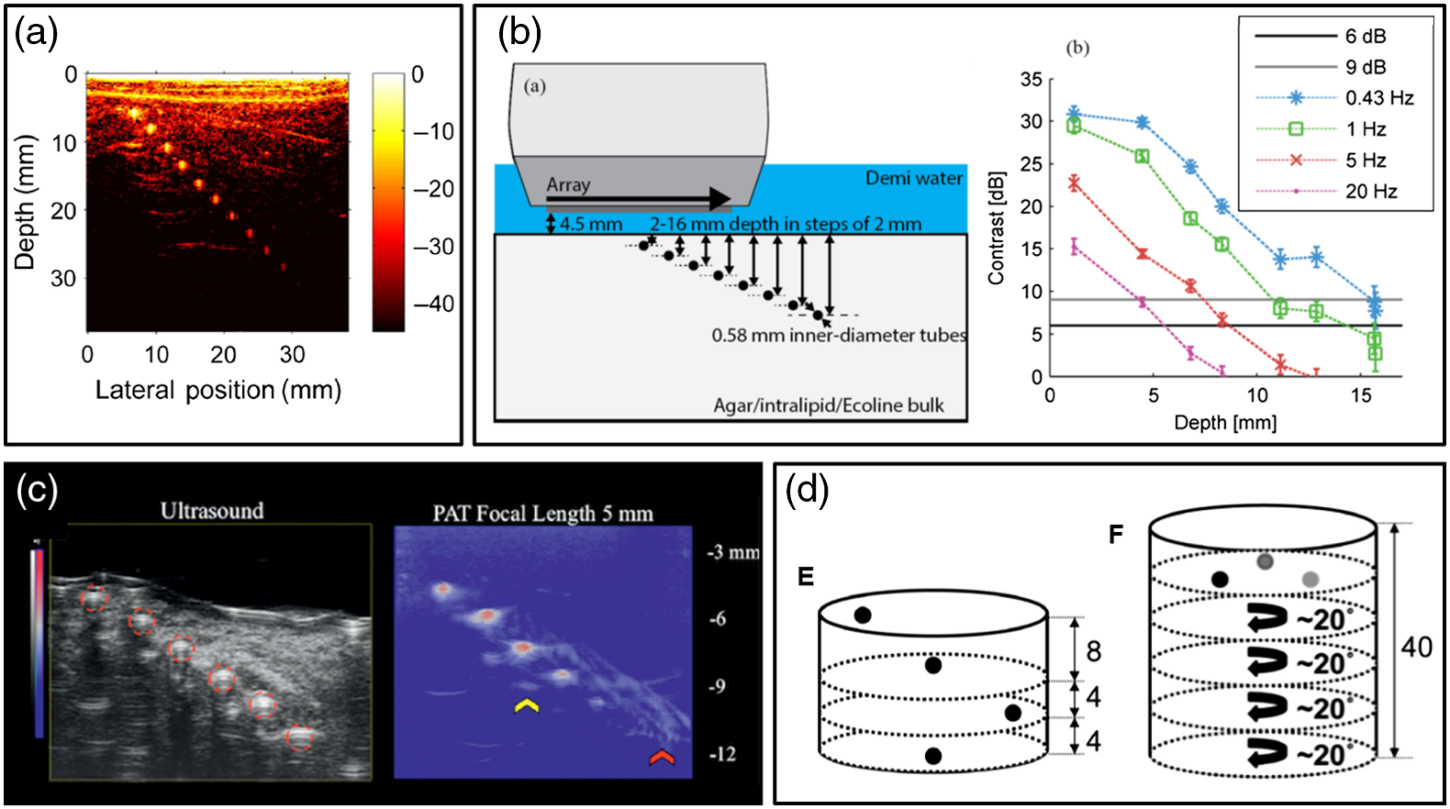
Representative approaches to evaluate photoacoustic image maximum depth of visualization. (a) PA image of a PVCP phantom containing a diagonal array of India ink-filled tubes. Reproduced and adapted with permission from Ref. 48. (b) Schematic of an array of black ink-filled polyethylene tubes in an agarose phantom, and the plot of contrast versus depth and frame rate. Reproduced and adapted with permission from Ref. 137. (c) Ultrasound and PA images of a PVA phantom embedded with six PE-50 tubes. Reproduced and adapted with permission from Ref. 53. (d) Schematic of turbid PVCP phantoms containing PVCP spheres with variable depth and absorption coefficient. Reproduced and adapted with permission from Ref. 83.

Depth of visualization was frequently evaluated in PAI phantom studies. The most common approach was to image a phantom containing an array of tubes placed at various depths, filled with relevant light-absorbing contrast media such as India ink, black dye, blood, or nanoparticles (Fig. 11).^48^^,^^80^^,^^128^^,^^137^^–^^139^ Alternative approaches included translating a single target to different depths in a liquid phantom^102^ or elevationally scanning the transducer over a phantom containing a vertically slanted tube^134^ or graphite sheet.^50^ Solid phantom inclusions were also used as imaging targets for depth testing such as black PVCP spheres in a PVCP background^83^ or polyurethane cylinders within polyurethane background.^58^ Some studies reported imaging depth based on detection of a target at one particular depth, which may underestimate maximum depth of visualization. While many studies focused on handheld epi-illumination PAI, one study tested depth of visualization for an endoscopic PAI device by placing 0.6-mm-diameter graphite rods at different radial positions in a cylindrical gelatin-milk phantom containing silica particles.^140^ Similar studies of imaging depth were performed for PAI systems using interstitial light sources placed within the phantom or tissue.^68^^,^^100^^,^^115^ These approaches demonstrate how the common diagonal tube array phantom design can be modified to suit different imaging system configurations.

In most studies, all targets had the same absorption coefficient, isolating the impact of target depth on detectability from the effect of target absorption variation (see Sec. 3.4). This approach was similar to the ultrasound penetration depth phantom shown in Fig. 5(b).^14^ However, some PAI studies have also varied absorption coefficient of the target array, which is somewhat similar to low-contrast detectability phantoms described in Sec. 2.4.^86^^,^^92^ Because depth of visualization depends on target absorption coefficient, target absorption values should be relevant to the intended imaging application and should include low-contrast conditions.

In addition to phantom design, there was wide variation in how, if at all, maximum depth of visualization was quantified. The details of how such metrics were computed from image ROIs (ROI size, shape, and location using average versus maximum values) were not always provided. Also, specifying a maximum imaging depth requires selection of an appropriate signal threshold. Some studies interpolated an image quality metric versus depth to find the crossover with a pre-specified threshold (e.g., SNR=2, or 6 dB), but others reported the depth of the deepest detectable target (even if the target appears well above the limit of detection). To determine maximum depth of visualization, there should be at least one target that is found to be undetectable such that maximum depth of visualization can be interpolated, as opposed to relying on extrapolation. To enable reproducibility, the methods of selecting ROIs and computing values from image data should always be comprehensively described.

**Fig. 12:**
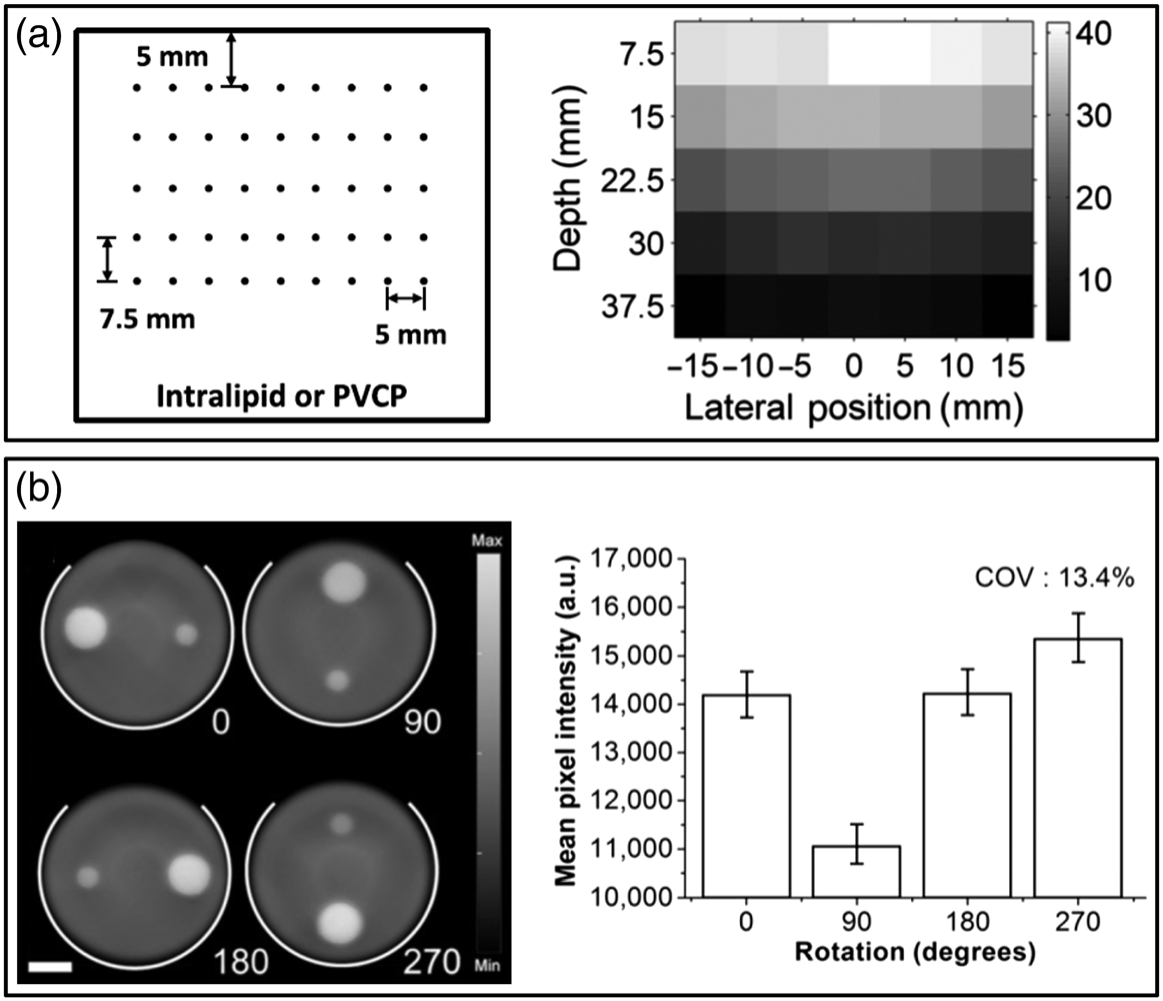
Representative approaches for evaluating photoacoustic image uniformity. (a) Schematic and resultant uniformity map of a PVCP phantom containing a steel wire grid. (b) PA images and computed mean target intensities for a polyurethane phantom containing absorptive targets imaged at 4 different rotations (0 deg, 90 deg, 180 deg, and 270 deg). Reproduced and adapted with permission from Refs. 48 and 57, respectively.

Image uniformity was evaluated much less frequently than depth of visualization, despite the close relationship between these IQCs. While standards measured uniformity in terms of variation in large, positive-contrast homogeneous regions, photoacoustic images generally do not present such features, e.g., due to boundary buildup effects. Thus, photoacoustic image uniformity may be more appropriately described by how the apparent brightness of an absorbing target varies within the field-of-view. Several studies measured SNR or contrast of high-contrast targets such as wires to characterize imaging depth or target detectability versus depth,^10^^,^^55^^,^^98^^,^^112^^,^^141^^,^^142^ but few studies evaluated uniformity in other dimensions (most notably, lateral uniformity). One approach measured 2D image uniformity in a turbid PVCP phantom containing an array of metal wires, plotting average target amplitude versus target position [Fig. 12(a)].^48^ Note that such wire or filament phantoms are often inappropriate for determining maximum depth of visualization owing to their high, non-biologically relevant absorption (unless the intended application involves detection of embedded manmade objects such as needles^143^ or brachytherapy seeds^68^). A few studies evaluated uniformity using larger inclusions with more moderate absorption levels, such as cylindrical absorptive inclusions in a turbid, acoustically attenuating polyurethane cylinder.^57^ This phantom was scanned in different angular positions and uniformity was determined as the variation in average target intensity with location in the field-of-view [Fig. 12(b)]. Another study measured variation in image intensity of methylene blue-filled tubes both laterally and with depth using a 3D-printed housing to control tube alignment and positioning.^139^

### 3.4 Sensitivity and Low-Contrast Detectability

**Fig. 13:**
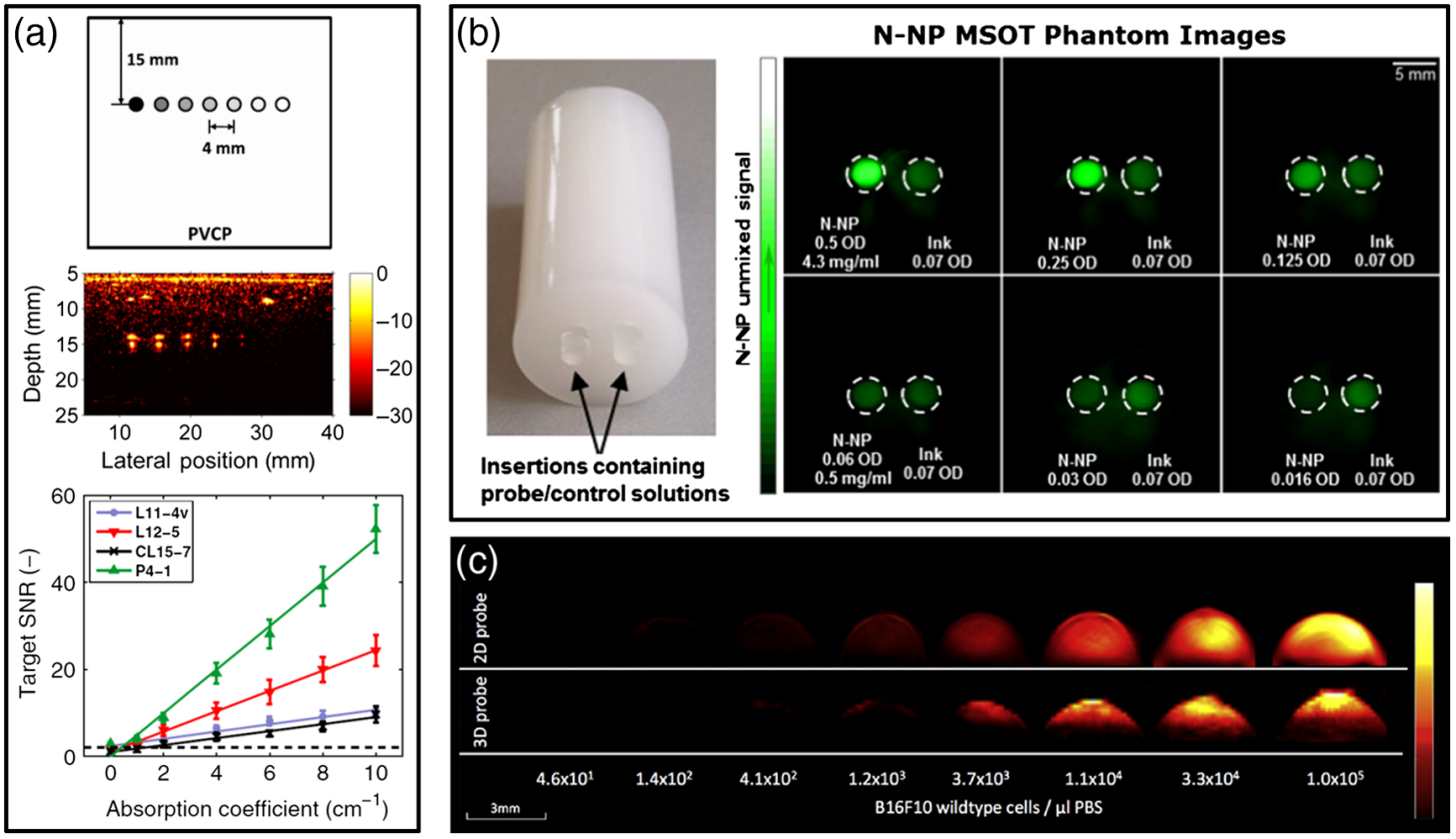
Representative approaches to evaluate photoacoustic image sensitivity. (a) Schematic and PA image of a PVCP phantom containing PTFE tubes filled with different concentrations of India ink, and plot of target pre-log compression SNR versus absorption coefficient for four transducers. (b) Photograph and PA images of an agar phantom with two cylindrical insertions filled with nanoparticles (P-NP) or a black ink solution. (c) PA images of agar plugs containing varying concentrations of B16F10 melanoma cells. Reproduced and adapted with permission from Refs. 11, 48, and 59, respectively.

Following medical imaging standards, we defined “sensitivity” testing as measurements of change in photoacoustic image amplitude versus target optical absorption or chromophore concentration to determine limits of detection. In some PAI articles, sensitivity referred to ultrasonic transducer sensitivity (e.g., responsivity in V/mPa or noise-equivalent pressure in Pa), rather than image sensitivity.^123^^,^^144^ Most sensitivity studies were performed to demonstrate detectability of exogenous contrast agents including dyes,^85^^,^^112^^,^^145^^–^^147^ encapsulated-ink microbubbles,^148^ and nanoparticles,^59^^,^^80^^,^^126^^,^^149^^–^^156^ although other studies evaluated endogenous chromophores, such as melanoma cells^11^^,^^157^ or blood with varying hematocrit.^128^ Some studies used generic absorptive targets such as embedded tubes^48^^,^^102^^,^^114^ or solid agar inclusions^158^ containing colored inks. The common approach was to generate a linear fit of measured image signal/intensity (in arbitrary units) versus target concentration or absorption. Target depth varied considerably from 1- to 2-cm depths to entirely superficial/exposed targets. Some phantoms contained several targets with varying absorption, whereas others sequentially filled the same inclusion with different absorptive solutions. Several studies used a commercial cylindrical polyurethane phantom containing two cylindrical insertions/chambers [similar to Fig. 13(b)].^145^^,^^146^^,^^149^ Most studies did not implement or propose a limit of detection based on these test data.

This general approach, while commonly used, has several limitations: First, presenting PAI amplitude in terms of arbitrary units prevents direct comparisons between studies. Assessing sensitivity using image quality metrics such as target CR or SNR may better facilitate performance comparisons across PAI systems. Second, establishing quantitative detection thresholds that agree with limits determined by visual inspection may be more practical and reproducible. Third, test results expressed in terms of contrast agent concentration may have limited utility. A more universal approach would be to use phantoms containing stable, well-characterized chromophores at well-defined absorption coefficients.^48^ It should then be possible to estimate results for different contrast agents if their molar extinction or absorption coefficients are known. Finally, most sensitivity phantoms contained targets of varying absorption strength but only at a fixed depth. The ideal phantom for testing sensitivity should have targets of various absorption coefficients located at several depths.^92^^,^^128^ It may also be appropriate to perform testing in phantoms with different background optical and/or acoustic properties to characterize how tissue background affects sensitivity and target detectability.^139^^,^^153^

While we identified several PAI sensitivity test methods, we did not find any low-contrast detectability phantom studies using various target sizes. This was surprising given the prevalence of such testing in medical imaging standards (Sec. 2.4). Target size may be expected to affect detectability in PAI, for instance due to differences in intra-target fluence distribution and out-of-plane signal contributions, as well as boundary buildup effects in larger targets. This is a significant current gap in available phantom-based performance methods for PAI. Suitable phantom designs may build on sensitivity and imaging depth phantoms, such turbid phantoms with arrays of targets of various absorption coefficient, placed at one or more depths.

### 3.5 Artifacts

**Fig. 14:**
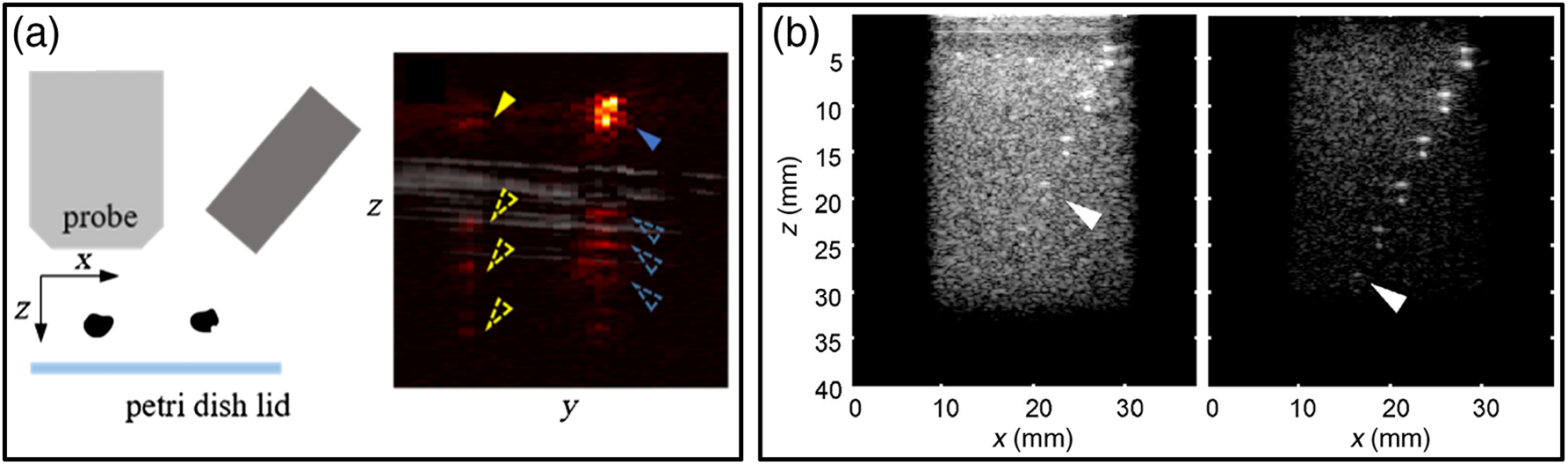
Representative approaches to evaluate photoacoustic image artifacts. (a) Diagram of an agarose phantom containing two black absorbers, one inside and one 3 to 4 outside of the image plane. An overlaid ultrasound/PA image shows resultant in-plane and out-of-plane artifacts. (b) PA images of a gelatin phantom containing 2-mm absorptive gelatin cylinders, generated using either conventional image reconstruction (left) or clutter reduction methods (right). Reproduced and adapted with permission from Refs. 138 and 166, respectively.

Photoacoustic images are susceptible to several well-known image artifacts including image clutter,^68^^,^^138^ reflection artifacts,^159^ out-of-plane artifacts,^48^^,^^160^ motion artifacts,^161^ scanning misalignment artifacts,^107^ boundary buildup,^162^ laser-induced electromagnetic interference,^163^ and limited view artifacts. Several studies used phantoms to evaluate performance of proposed correction techniques for specific types of artifacts. One study used a SMOFLipid-agar phantom containing 0.7-mm diameter graphite rods to evaluate reduction of x-shaped reconstruction artifacts using dynamic focusing and coherence weighting.^123^ Another study evaluated a technique to remove reflection artifacts caused by acoustic heterogeneity using a clear gelatin phantom^164^ or water bath^165^ containing inclusions with different acoustic properties from the background medium. Artifact reduction was quantified using intensity reduction ratio, i.e., the ratio of original to corrected ROI intensity. Two articles by Nguyen and Steenbergen^160^ and Nguyen et al.^167^ described phantom-based evaluation of out-of-plane artifacts caused by photoacoustic signals from absorbers near the imaging plane [Fig. 14(a)]. These studies involved either transparent agarose phantoms or Intralipid solutions containing pairs of absorbers such as short lengths of sub-millimeter black threads or sutures. Phantoms either had inclusions at the same depth or positioned the out-of-plane absorber at a shallower depth in order to cause direct overlap of image artifacts with the in-plane target. One of these studies defined artifact-to-noise ratio, the mean artifact ROI amplitude divided by mean background ROI amplitude.^160^ In another study, an acoustic radiation force technique for reducing photoacoustic image clutter was evaluated using gelatin phantoms doped with ${\mathrm{TiO}}_{2}$, India ink, and cellulose, and containing an array of tubes at different depths [Fig. 14(b)].^138^ Clutter reduction was evaluated in terms of improved SNR and maximum depth of visualization (see Sec. 3.3). A similar approach used a gelatin-cellulose phantom but quantified clutter reduction using target SBR.^84^ While not all studies quantified artifact strength or reduction efficacy, most that did compared contrast-based image quality metrics, rather than noise-based metrics.

Due to the wide variation in PAI artifacts and how they impact performance, it may be difficult to develop a single phantom to quantitatively assess all possible artifacts. As with medical imaging standards, future consensus test methods may need to be tailored to individual artifacts. Still, we recommend establishment of general best practices for assessing PAI artifacts, such as use of biologically relevant phantoms that replicate artifacts of interest and establishment of well-defined metrics to quantify artifacts.

## 4 Discussion and Outlook

We reviewed 32 consensus documents and standards for established medical imaging modalities as well as nearly 120 PAI articles describing phantom-based image quality test methods. Our review of test methods for ultrasound, CT, x-ray mammography, and MRI revealed similarities and differences in terms of IQCs, phantom geometries, TMM properties, data acquisition and analysis procedures, and the level of prescribed detail for different aspects of testing. Insights gained from this review have the potential to facilitate standardization, clinical translation, and the maturation of PAI into a well-accepted medical imaging modality.

The most common IQCs used in medical imaging standards were in-plane spatial resolution, out-of-plane spatial resolution (slice thickness), geometric accuracy, image uniformity, depth of visualization, sensitivity, and low-contrast detectability. These IQCs should be considered in the development of PAI standards, as well as others that address key aspects of image quality including distortion, artifacts, and co-registration accuracy. Unlike medical imaging standards, PAI literature focused on a smaller number of IQCs (e.g., in-plane resolution, depth of visualization, and sensitivity). It is possible that developers would elect to test more IQCs if the burden of developing and validating suitable test methods were reduced through phantom development and commercialization. Some of the understudied IQCs for PAI are linked to well-known device challenges: elevational resolution is often poor for linear array transducers and relates to out-of-plane artifacts; geometric accuracy, distortion, and co-registration accuracy relate to image reconstruction algorithm performance; and image uniformity and depth of visualization relate to fluence distribution. While it is important to ensure that a sufficient range of IQCs are tested to adequately characterize performance, PAI standards will need to balance this consideration against the potential for creating excessive burdens for developers and users. Achieving this balance could be accomplished, in part, by recommending the use of fewer IQCs and simpler test methods in roles such as post-market QC and constancy testing, whereas more extensive and rigorous testing would be reserved for device development, performance verification, and regulatory evaluation.

Tissue-simulating phantoms were critical components of nearly all image quality standards. These standards tended to implement relatively simple designs for objective, quantitative assessment of image quality, such as homogeneous regions with simple inclusions in repeating patterns. Phantom properties tended to be relevant to generic tissue, rather than matching a specific tissue type. While standards often specified required phantom material properties and geometry, they generally did not mandate a particular material for background regions or inclusions (although in some cases, suitable examples were mentioned). In principle, any TMM meeting test method requirements and relevant to the imaging application would thus be acceptable. But to maximize consistency in test results, future PAI standards may elect to identify a preferred TMM and allow other options if they are shown to generate identical test results. Also, most accreditation programs required use of specifically approved commercial phantoms that have been rigorously characterized by the manufacturer to ensure conformity to standards during acceptance testing, QC, and maintenance/repairs. Some of these phantoms are also traceable to gold standard metrology, such as those supported by the National Institute of Standards and Technology (NIST).^168^ This may be an important future consideration for PAI standards, especially for quantitative imaging applications, and is an active area of development in biophotonics.^169^^,^^170^

It should be stressed that while appropriate TMMs are essential for phantom-based test methods and the community is actively working toward addressing this need, careful design and consistent reproduction of phantom geometry, target inclusion sizes and patterns, and measurement/analysis protocols is equally important. Image quality standards often provided detailed, yet relatively simple, test protocols that specified ROI dimensions and locations, number of images to acquire, and explicit formulas for computing image quality metrics. Standards also often recommended using a fixed set of application-relevant image processing and display settings for a given test. While some variation in nomenclature and definition of image quality metrics was seen across medical imaging standards, we observed much broader variation in definitions for photoacoustic image quality metrics such as SNR, SBR, CR, and CNR. Future PAI standards should explicitly define recommended image quality metrics, and one self-consistent set of metric definitions would be $S=S/\sigma_{B}$, CR=SBR=S/B, and $\mathrm{CNR}=(S-B)/\sigma_{B}$. Data acquisition procedures, image analysis methods, and image quality metrics should always be comprehensively described to ensure test reproducibility. It is notable that some test methods involved subjective image evaluation by a reader. While there is certainly value to such an approach as it mirrors how images will be used clinically, objective methods are typically preferred to maximize repeatability and reproducibility. Standards were often not accompanied by minimum acceptance criteria. While PAI studies generally have not attempted to establish minimum performance thresholds, such criteria may be useful for devices that focus on specific applications, such as breast cancer detection. In the development of PAI standards, it will be critical that procedures for data acquisition, image analysis, and metric calculation are comprehensively described, so as to optimize reliability of comparisons between tests performed by different groups. While this review has focused primarily on image quality standards, additional standardized test methods will be needed for quantitative and functional PAI biomarkers such as blood oxygen saturation. These tests will likely require the use of specific materials such as blood or contrast agents incorporated within inclusions of a larger tissue-simulating phantom.^6^^,^^171^ Also, while not typically addressed in standards, future consensus test methods focusing on tissue-specific device applications may benefit from biomimetic, anthropomorphic phantoms to provide more clinically realistic, task-based image quality assessment approaches.^172^^–^^174^

Many of the issues addressed in this review apply to the standardization of other existing and emerging biophotonic approaches. Some IQCs mentioned here have been addressed in endoscopy performance standards,^34^ but may also be relevant to more advanced biophotonic modalities such as optical coherence tomography^175^ or diffuse optical imaging.^176^ Insights from this review on phantom design and test methodology may inform standards development in both sub-surface, cross-sectional optical imaging modalities (e.g., diffuse optical imaging/tomography, fluorescence tomography, and optical coherence tomography) and superficial, *en face* modalities (e.g., fluorescence, hyperspectral, and Raman imaging).

## 5 Conclusion

As the photoacoustics community and others within the field of biomedical optics work toward establishing consensus standards, available medical imaging standards should be consulted. These documents can facilitate and accelerate establishment of best practices for photoacoustic image quality assessment. The past decade has seen significant advances in TMM development for PAI, but more progress is needed on this topic and in development of standard image acquisition and data analysis protocols. Further work is also needed to expand and adapt existing phantom test methods into multiple variations that are useful for the broad range of PAI device configurations reported in the literature. These efforts should culminate in establishment of a PAI performance standard, which will mark a key milestone in the maturation of this technology. Such consensus documents have the potential to accelerate device development and optimization, minimize duplication of effort, and facilitate clinical translation.
